# The Bud App: A protocol for a randomized controlled trial with an internal pilot phase targeting modifiable risk and protective factors for suicide among international students

**DOI:** 10.1016/j.conctc.2025.101578

**Published:** 2025-11-26

**Authors:** Samuel McKay, Christina Ng, Jennifer Nicholas, Vivienne Browne, Gina Chinnery, Isabella Choi, Bailey Nation-Ingle, Kristal Alison, Ella Perlow, Michelle Lamblin, Elise Carrotte, Ellie Brown, Gregory Armstrong, Jocelyn I. Meza, Madhavan Mani, Jo Robinson

**Affiliations:** a Orygen, Parkville, Australia; b Centre for Youth Mental Health, The University of Melbourne, Melbourne, Australia; c Sydney Medical School, Faculty of Medicine and Health, The University of Sydney, Sydney, NSW, Australia; d Suicide Prevention and Response Office, Victorian Department of Health, Victorian Government, Australia; e Australian Research Council's Centre of Excellence for Children and Families Over the Life Course, Australian Research Council, Melbourne Institute, Melbourne, Australia; f Centre for Mental Health and Community Wellbeing, Melbourne School of Population and Global Health, The University of Melbourne, Melbourne, Australia; g Department of Psychiatry and Biobehavioral Sciences, University of California, Los Angeles, USA; h Centre for Digital Transformation of Health, Faculty of Medicine, Dentistry and Health Sciences, The University of Melbourne, Melbourne, Australia

**Keywords:** International students, Suicide prevention, Digital interventions, Mental health

## Abstract

International students face elevated risk for mental health problems and suicide, yet encounter multiple barriers to accessing timely and culturally responsive treatments. Key challenges include high rates of psychological distress, low mental health literacy, and reduced help-seeking, often compounded by perceived burdensomeness, a lack of belonging, and difficulties with emotion regulation. Bud is a self-guided, co-designed mobile app developed to address these challenges through an accessible digital intervention. This paper outlines a protocol for a randomized controlled trial evaluating the effectiveness, acceptability, engagement, and safety of Bud among international students enrolled at Australian tertiary institutions. The trial also includes an internal pilot phase to ensure the feasibility of the trial procedures, with benchmarks for recruitment, retention, and engagement. A community sample of 302 participants will be randomly assigned to either the intervention group (Bud app) or the active control group (online mental health fact sheets) for four weeks. Assessments will be completed online at baseline, two weeks, and four weeks post-baseline. The primary outcome is psychological distress, with secondary outcomes including help-seeking intentions, perceived burdensomeness and belonging, emotion regulation, mental health literacy, and suicidal ideation. Acceptability and engagement will be assessed using self-report and objective measures. Interviews will be conducted with a subset of 20 participants to explore their views on Bud further. This trial will provide the first evaluation of a suicide prevention tool specifically for international students. If effective, Bud could offer a scalable and culturally relevant solution to improve student mental health and reduce service access barriers.

**Trial registration:**

ACTRN12625000584437.

## 1 Background

Suicide is a leading cause of death for young people aged 15–29 [1], and rates are particularly elevated in students enrolled in tertiary education, such as university or college [2]. International students comprise a significant proportion of tertiary education populations globally [3], yet they face unique challenges that can adversely affect their mental health [4]. Although past-year rates of suicidal ideation and self-harm are similar between international and domestic students (5.6–9.8 % vs. 5.2–13.3 % for ideation; 4.3–17.2 % vs. 3.2–22.9 % for self-harm), international students report higher rates of suicide attempts (1.21–2.2 % vs. 0.1–1.6 %) [5]. This elevated risk for severe outcomes appears to be driven by a complex interplay of stressors, including academic pressure, acculturative stress, and stigma [5].

Despite growing awareness of these risks, most suicide prevention still depends on traditional support, such as clinical treatment through health services or generic mental health information delivered universally by educational institutions [6]. There are two key limitations to these standard approaches. First, international students, including those who die by suicide, are less likely to engage with health services because of barriers such as stigma, visa concerns, low mental health literacy, and high treatment costs [[7], [8], [9], [10]]. Second, students who seek support often find services difficult to access when needed, poorly adapted to their cultural context, or mismatched to their expectations, leading to disengagement [11,12]. These findings highlight a clear need for suicide prevention strategies that are developed *with* and *for* international students and delivered in ways that are timely, relevant, and accessible [13].

Digital interventions targeting modifiable risk and protective factors for suicide represent a promising avenue for suicide prevention with international students [13,14]. They can offer scalable, low-cost, and on-demand support that overcomes key barriers to help-seeking and treatment utilization, particularly for culturally and linguistically diverse populations [14,15]. Evidence suggests that digital programs can improve mental health literacy, reduce stigma, enhance self-regulation skills, and, in some cases, directly reduce suicidal ideation [15,16].

A growing number of such programs have demonstrated short-term benefits in university settings. Universal interventions, such as online gatekeeper training (e.g., *ASK About Suicide*, *Kognito*), improve suicide-related knowledge and preparedness but have shown mixed effects on behaviour and are rarely tailored to the needs of diverse student groups [17,18]. Selective and indicated interventions like *eBridge* and *LifeBuoy* have demonstrated improvements in help-seeking and reductions in suicidal ideation among students with elevated risk [19,20]. However, existing programs have largely overlooked international students, despite their heightened vulnerability and well-documented barriers to engaging with support services [6].

We sought to address this gap through the development of *Bud*, a co-designed mobile app grounded in evidence-based therapeutic approaches and informed by best-practice recommendations for proactive engagement and risk screening [6]. Developed in collaboration with international students, the app is designed to be accessible, non-stigmatizing, and aligned with the common experiences and challenges faced by this population, such as academic pressure, social isolation, and acculturative stress [21,22]. Cultural responsiveness was embedded through a structured co-design process with international students, ensuring that the app's content, tone, and delivery reflected their lived experiences, preferences, and values. This included avoiding clinical language, incorporating personal narratives, and designing flexible, self-guided tools that align with students' needs and challenges.

Informed by this process, Bud functions as a community-level intervention that targets modifiable risk and protective factors associated with suicide through a suite of brief, interactive tools, personal stories, and skills-based modules. Rather than addressing a single diagnosis, *Bud* adopts a transdiagnostic approach by targeting shared mechanisms that underlie psychological distress, suicidality, and related mental health challenges [23,24]. At its core is an emotion-tracking feature that facilitates emotional self-awareness and recommends contextually appropriate support tools, consistent with the principles of just-in-time adaptive interventions [25]*.* The tools draw on cognitive behavioural (CBT), dialectical behavioural (DBT), and acceptance and commitment (ACT) therapies to reduce psychological distress, strengthen emotion regulation, and support values-based coping strategies associated with reduced suicidal ideation [16,26].

To address interpersonal risk factors such as perceived burdensomeness and thwarted belonging [27]*,* the app includes activities informed by evidence-based strategies such as self-compassion, values reflection, and small social actions that promote connection [28]*.* Help-seeking is indirectly supported through stories from international students sharing their lived experiences of studying, living abroad, and navigating suicidal thoughts. These narratives aim to normalise help-seeking and validate users’ emotional experiences. Evidence suggests that suicide prevention narratives featuring peer experiences of coping and recovery can increase help-seeking intentions, reduce suicidal ideation, and foster identification with the storyteller, particularly among young people [29]*.* Additionally, Bud features a brief, peer-support-focused gatekeeper module aligned with non-Western adaptations [30,31] that equips students with the skills to recognise potential suicidal ideation in others and respond effectively. This component was developed in response to co-design feedback indicating that while many students were reluctant to engage with suicide-specific content for themselves, they were open to learning how to help others.

The selection of outcomes for this trial was guided by Bud's theory of change and its positioning as a community-level suicide prevention intervention. Designed to support international students across the continuum of psychological distress, Bud delivers evidence-based skills that address upstream risk and protective factors for suicide, offering early intervention for those not yet experiencing suicidal thoughts, and meaningful support for those who are. Accordingly, psychological distress was selected as the primary outcome due to its high prevalence in this population (with approximately 50 % reporting moderate to severe distress) [32,33], its robust association with suicidal thoughts and behaviours [34], and its demonstrated responsiveness to low-intensity self-guided digital interventions [35,36].

The secondary outcomes were selected to capture key suicide-related risk and protective factors targeted by the intervention. Help-seeking intentions were assessed due to consistent evidence that international students, including those who die by suicide, are often reluctant to engage with mental health services [8,9]. Perceived burdensomeness and sense of belonging were included based on their established associations with suicidality [37,38] and high relevance to international students, who frequently report experiences of academic underperformance, isolation, and cultural disconnection [4,5]. Suicidal ideation was included as a core suicide-related outcome to assess the app's impact on direct indicators of suicide risk. Mental health literacy was included because it supports symptom recognition, self-care, and help-seeking, and is often lower among international students compared to domestic peers [9,39]. Emotion regulation was included as a hypothesised mechanism of change, given its established link to reduced suicidal thoughts and behaviours [40]. This construct is targeted through several app features, including the emotion tracking and recommendation system, which aim to strengthen students' capacity to manage distress in the moment.

This study will evaluate the effectiveness, acceptability, engagement, and safety of Bud, a co-designed digital suicide prevention app for international students (see Table 1 for a summary of the study aims and hypotheses). The primary aim is to determine whether Bud leads to greater reductions in psychological distress compared to an active control condition. The secondary aims include evaluating whether Bud leads to improvements in suicide-related risk and protective factors, including help-seeking intentions, perceived burdensomeness, sense of belonging, emotion regulation, and suicidal ideation. The study will also assess the app's acceptability, monitor for any iatrogenic effects, and evaluate user engagement using self-report and app-derived metrics. An embedded internal pilot phase will assess the feasibility of trial procedures, with predefined benchmarks for recruitment, retention, and intervention engagement. Interviews will be conducted with a subset of participants to further explore their views on Bud's acceptability, usability, and opportunities for implementation.

**Table 1:** Study aims and hypotheses.

Aims	Hypotheses
**Aim 1:** To evaluate the effectiveness of *Bud* compared to an active control condition on psychological distress and suicide-related risk and protective factors	***Primary outcome***
	*H1:* Participants using *Bud* will report greater reductions in psychological distress compared to the control group at post-intervention.
	***Secondary outcomes***
	*H2:* Participants using Bud will report greater increases in help-seeking intentions compared to the control group at post-intervention.
	*H3:* Participants using *Bud* will report greater reductions in perceived burdensomeness and thwarted belongingness compared to the control group at post-intervention.
	*H4:* Participants using *Bud* will report greater improvements in emotion regulation compared to the control group at post-intervention.
	*H5:* Participants using *Bud* will report greater increases in mental health literacy from the intervention than the control group.
	*H6:* Participants using *Bud* will report greater reductions in suicidal ideation compared to the control group.
**Aim 2:** To assess the acceptability of the Bud app among international student users	*H7:* Participants in the Bud group will rate the app as acceptable, including positive evaluations of its engagement, functionality, aesthetics, information quality, and overall subjective quality.
**Aim 3:** To assess engagement with the Bud app	*H8:* Participants in the Bud group will demonstrate high engagement, reflected in app usage metrics and perceived helpfulness of activities.
**Aim 4:** To assess the safety of the Bud app	*H9:* No iatrogenic effects (e.g., increases in suicidal ideation or psychological distress) will be observed or reported among participants in the Bud group.

## 2 Methods

### 2.1 Overall trial design

This project employs an open-label, parallel-group randomized controlled trial (RCT) stratified by suicidal ideation to evaluate the effectiveness of the Bud app intervention. The trial will be conducted in accordance with the CONSORT (CONsolidated Standards Of Reporting Trials) 2025 Statement. Participants (N = 302) will be randomly allocated to either the Intervention group (Bud app; n = 151) or the active Control group (mental health factsheets; n = 151). Intervention group participants will have access to the Bud app content and features for four weeks. They will be encouraged to use it at least once per week, but may engage with it as much or as little as they choose. Control group participants will receive access to four mental health fact sheets over the same timeframe. All content in both conditions will be delivered in English. Assessments will occur at baseline (T1), two weeks post-baseline (T2), and post-intervention (four weeks post-baseline; T3), enabling the analysis of intervention effects both within and between groups over time.

### 2.2 Sample size

The target sample size of 302 participants (151 per group) was determined through an a priori power analysis conducted using the SIMR package in R [41]. We simulated 1000 datasets for a 2 (intervention vs. control) × 3 (T1, T2, T3) repeated-measures linear mixed-effects model—with fixed effects for group, time, and their interaction, random intercepts, moderate correlations, and a nonsphericity correction factor of 1. Specifying a moderate Group × Time interaction (f ≈ 0.25) at α = 0.05, we found that 252 participants (126 per group) would yield ∼80 % power; inflating for an anticipated 20 % attrition rate resulted in a final sample target of N = 302 (151 per group).

### 2.3 Study sites

The study will be led by researchers at Orygen, the Centre for Youth Mental Health (CYMH) at the University of Melbourne. However, there are no specific study sites, as participants will be from a community sample and will access the interventions remotely using their mobile phones. Both interventions are designed to be used flexibly, allowing participants to engage at times and locations that are convenient for them. All study data will be collected through online surveys administered via REDCap.

### 2.4 Participants

#### 2.4.1 Inclusion criteria

To be eligible for the study, participants must be international students currently enrolled at an Australian tertiary education institution and residing onshore in Australia. They must be 18 years or older, able to read and speak English and have access to a smartphone running either Android or iOS. Suicidal ideation was not required for inclusion, as Bud is designed as a community-level, upstream suicide prevention program for international students.

#### 2.4.2 Exclusion criteria

There are no predefined exclusion criteria for the study. However, a safety management protocol is in place. Participants who indicate suicide risk during surveys, request a phone check-in, and are assessed as being at imminent risk of harm during the screening call will be withdrawn from the study and referred to appropriate support services. Participants with ongoing suicidal ideation who do not report imminent risk may remain in the study.

### 2.5 Comparators

Participants will be randomly assigned to the Bud App (intervention) or mental health fact sheets (control) group.

#### 2.5.1 Intervention condition: Bud App

The Bud App is a self-guided digital intervention to support international students’ mental health and wellbeing. It targets modifiable risk factors, such as stress and social isolation, while strengthening protective factors, including social support and help-seeking behaviours. The app is intended for use across the continuum of psychological distress, from mild stress to heightened emotional states, but is not a crisis intervention tool. Instead, it provides timely, low-intensity support and directs users to crisis services when needed.

Bud was co-designed with international students to ensure that the app reflects the shared challenges and preferences of this population. While not formally adapted for specific cultural or linguistic groups due to the diversity within international student populations, Bud incorporates culturally sensitive design features, such as non-clinical framing (e.g., no use of terms like “diagnosis” or “therapy”), diverse student narratives, and peer-oriented content to reduce stigma and promote accessibility across a range of cultural backgrounds.

Bud's design is grounded in just-in-time adaptive intervention principles [25]. An emotion-tracking feature promotes emotional awareness and recommends contextually relevant coping tools. Core therapeutic content is based on ACT, CBT and DBT approaches, such as reframing cognitive distortions, using temperature-based distress tolerance techniques, practising gratitude, guided breathing techniques, and structured problem-solving [42,43]. The app also includes a brief suicide prevention gatekeeper course, designed to equip users with the skills to recognise and respond to signs of suicide risk in themselves and their peers, aligned with best-practice adaptations for non-Western contexts [30,31].

To normalise help-seeking and reduce mental health stigma, the app also features stories from international students who share their experiences of navigating mental health challenges and seeking support [44]. Lastly, a gamified element is included, where users earn points that contribute to the growth of their virtual ‘Bud’ character. Participants are free to engage with the app in a manner that suits their individual preferences, with optional daily mood check-in reminders through push notifications and flexible access to content.

#### 2.5.2 Control condition: Mental health fact sheets

Participants assigned to the control group will receive access to four online psychoeducational fact sheets that provide general mental health information tailored for international students. These fact sheets were developed by a registered psychologist with experience in mental health communications and are representative of resources typically available on university websites and tertiary education platforms. The topics covered in the fact sheets include strategies for staying well through self-care, building and maintaining social connections, recognising the signs that indicate the need for mental health support, and information on how to access professional mental health services. Each fact sheet is designed to take approximately 5 min to read. All fact sheets will be available to participants, but they will be encouraged to read one fact sheet per week. Participants will receive weekly text reminders during the four-week study to access any fact sheets they haven't yet viewed. This flexible format lets participants engage with the content at their own pace and on their own schedule.

While this control condition was designed to reflect typical online supports provided to international students (e.g., psychoeducational materials), this represents a lower level of interactivity than the Bud app. This approach was selected to mirror “usual care” and to assess whether Bud offers additional benefit beyond the type of digital resources students commonly receive. A more technologically matched control could be considered in future research to isolate the effects of the intervention further. However, these options were beyond the current project's scope and resources.

### 2.6 Recruitment

Participants will be recruited via digital communication channels serving international students, including student email lists, social media pages for international students in Australia, and institutional communication platforms, over 12 months. Partnerships with tertiary education institutions (e.g., University of Melbourne) and organisations (e.g. Study Melbourne) working with international students in Australia will facilitate recruitment. Recruitment materials will invite students to complete a brief online pre-screening survey hosted in REDCap, which includes eligibility questions confirming international student status (“Are you an international student currently studying at an Australian university?”), age (≥18 years), English language proficiency, and access to a smartphone (Android or iOS). Student status will therefore be verified through self-report, consistent with prior large-scale digital trials [19,20]. Interested students will access study information via a Participant Information and Consent Form (PICF) hosted on REDCap.

### 2.7 Randomization

Participants will be randomly assigned to either the intervention or control group using a stratified block randomization approach with a block size of six. Stratification will be based on participants’ baseline suicidal ideation (SI) levels, as measured by the YSIS-3 [45], to ensure balanced allocation across three predefined strata: no or mild SI (scores 0–3), moderate SI (scores 4–6), and severe SI (scores 7 and above). The randomization sequence will be generated using a R and implemented within REDCap by a researcher independent of participant management, ensuring allocation concealment until assignment. Due to the nature of the intervention, participants cannot be blinded to their group allocation.

### 2.8 Retention

Follow-up surveys will be emailed two weeks (T2) and four weeks (T3) post-baseline. To support retention, participants will receive up to three reminder emails for each follow-up survey. At each time point, participants will have a defined window to complete the survey (seven days for T2 and two weeks for T3). Participants will be compensated $15 for each completed survey via direct transfer, with payment details collected at baseline. All participants will be encouraged to complete follow-up surveys regardless of their engagement with the intervention or control materials to support an intention-to-treat analysis.

### 2.9 Assessments

Assessments will be conducted remotely via REDCap (Research Electronic Data Capture [46,47]) at T1, T2, and T3 (see Table 2).

A. *Descriptive Information.* Demographic variables collected at baseline (T1) will include age, gender, country of origin, time in Australia, nationality, education level, study area, degree level, self-rated English language proficiency, and current grade point average (GPA). In addition, participants will be asked to report on previous suicide attempts and considerations of attempts (past 12 months), self-harm behaviours (past 12 months), adjustment to university (College Adjustment Questionnaire: CAQ; [48]), and adverse childhood experiences (Adverse Childhood Experiences Checklist: ACEs [49]).
B. *Outcomes.* Primary and secondary outcomes will be assessed at T1, T2 and T3.
  The primary outcome of this study is psychological distress measured with the Kessler Psychological Distress Scale (K10) [50]. This measure has been validated in diverse populations and is widely used in health related research [56]. Secondary outcomes will include key risk and protective factors for suicidal thoughts and behaviours targeted by the app, including help-seeking intentions using the General Help-Seeking Questionnaire (GHSQ [51]); Perceived burdensomeness and belonging using the Interpersonal Needs Questionnaire (INQ [52]); Emotion regulation using the Difficulties in Emotion Regulation Scale Short Form (DERS-SF [53]); Suicidal ideation using the Youth Suicide Ideation Screen–3 (YSIS-3 [57]); and mental health literacy with the Mental Health Learning Scale (MHL-9 [54]) adapted to refer to the interventions rather than school settings. All instruments are brief psychometrically validated tools designed for self-reporting and appropriate for digital intervention studies.
C. *Acceptability and Iatrogenic effects.* Acceptability of the intervention will be assessed at T3 in the intervention group using the User Version of the Mobile Application Rating Scale (uMARS [55]) and a single general satisfaction item (e.g., how satisfied were you with the app?). To assess both acceptability and potential iatrogenic effects, participants in both trial arms will complete a custom measure previously used in suicide prevention research [58]. This measure captures perceived acceptability (e.g., whether the app or fact sheets were enjoyable or worthwhile) and potential iatrogenic effects (e.g., whether the content was upsetting or increased suicidal thoughts). Changes in suicidal ideation, along with monitoring of adverse events, will also provide additional insight into potential iatrogenic effects.
D. *Engagement.* For the control intervention, the number of times the fact sheets are accessed will be recorded via REDCap. For the Bud intervention group, app usage data will be collected from participants to assess engagement and perceived usefulness of the Bud app. Specifically, this will include the total number of times the app is accessed, the number and type of activities completed, and responses to in-app prompts asking whether each activity was helpful. These metrics will quantify overall dosage and identify which features are most frequently accessed and perceived as most useful by participants. Participants will also have the opportunity to express interest in participating in an interview about their experience with the app.
E. *Interview questions.* A subset of participants from the Bud intervention group who express interest will be randomly selected and invited to participate in online qualitative interviews conducted on Microsoft Teams. We will aim to recruit 20 participants. They will receive $30 for participating in an interview and will be asked to reflect on the app's design, usability, technical challenges encountered, and privacy and security concerns related to sharing personal information within the app. Additionally, the interview will explore the app's content regarding cultural appropriateness, relevance to their experiences as international students, their thoughts about how it could best be disseminated to international student populations, and suggestions for improvement.

**Table 2:** Study measures at each time point.

Domain	Measure	# items	T1	T2	T3
Descriptive information	Demographic questionnaire	14	X		
	Previous Suicide attempts	2	X		
	Previous self-harm behaviours	1	X		
	Adjustment to University: CAQ [48]	14	X		
	Adverse Childhood Experiences: ACEs [49]	11	X		
Primary outcome	Psychological Distress: K10 (50)	10	X	X	X
Secondary outcomes	Help-Seeking: GHSQ [51]	12	X	X	X
	Perceived Burdensomeness: INQ [52]	5	X	X	X
	Sense of Belonging: INQ [52]	5	X	X	X
	Emotion Regulation: DERS – SF [53]	18	X	X	X
	Suicidal ideation: YSIS-3 (45)	3	X	X	X
	Mental health literacy: MHLS-9 (54)	9			X
Acceptability & iatrogenic effects	Open feedback question	1	X	X	X
	Acceptability and Iatrogenic effects: Custom Measure	6			X
	***Intervention group only***				
	Acceptability: Funder required questions	2			X
	Acceptability of app: uMARS [55]	20			X
	Interview invitation	1			X
Engagement metrics	***Control group only***	Web metrics		X	X
	Number of times factsheets accessed				
	***Intervention group only***				
	App usage (number and time accessing)	App metric		X	X
	Number and type of activities completed	App metric		X	X
	Perceive helpfulness of activities	In-app self-report		X	X
Total # of survey items per group			I = 96	I = 54	I = 92
			C = 96	C = 54	C = 69

Note: I = Intervention group, C = Control group. Each measure is described in detail below.

### 2.10 Data analysis

#### 2.10.1 Internal pilot phase

An internal pilot phase assessing feasibility will be conducted during the first three months of recruitment, using a Red-Amber-Green (RAG) rating system to evaluate key trial feasibility outcomes: recruitment, retention, and engagement [59]. This structured framework enables early identification of challenges and supports timely adaptations to ensure trial progression, including stopping actions for the trial if thresholds are not met. Importantly, no primary or secondary outcome data will be reviewed during this phase, preserving trial integrity and allowing full use of all collected data if feasibility benchmarks are achieved. The thresholds and associated actions are outlined in Table 3.

**Table 3:** Feasibility assessment thresholds and actions.

Domain	Red (Significant Barrier – Immediate Action Required)	Amber (Needs Attention – Adaptive Action Recommended)	Green (On Track – No Action Required)
**Recruitment**	<30 % of target sample recruited within 6 months despite active efforts.	30–50 % recruited within 6 months.	≥50 % recruited within 6 months.
	*Action:* Review study viability with sponsor and HREC; consider pausing to revise recruitment strategy or halting trial.	*Action:* Expand recruitment channels, revise outreach materials, increase institutional partnerships.	
**Retention**	<40 % of participants complete follow-up surveys.	40–60 % complete follow-up surveys.	≥60 % complete follow-up surveys.
	*Action:* Investigate barriers to retention; assess feasibility of continuing; consider additional supports or modified data collection.	*Action:* Use additional automated reminders, explore additional incentives, assess survey burden.	
**Engagement**	<30 % of participants use the app at least once.	30–50 % use the app at least three times.	≥50 % use the app at least three times.
	*Action:* Conduct urgent review of app usability and content; consult with developers; assess feasibility of updates within study timeframe.	*Action:* Explore opportunities for additional push notifications, adjust onboarding, enhance motivational elements, or enhance UX elements.	

#### 2.10.2 Main analyses

Changes in the primary and secondary outcomes will be evaluated using generalised linear mixed models (GLMMs), including random intercepts for participants, random slopes for time, and fixed effects for group, time, and their interaction. For zero-inflated outcomes such as suicidal ideation (e.g., a large number of individuals reporting no ideation), model diagnostics will be used to assess the appropriateness of the GLMM approach, with consideration of alternative specifications (e.g., two-part models such as the zero-inflated Poisson model) if indicated [60,61]. Measures of acceptability and iatrogenic effects will be assessed with descriptive statistics. Pearson correlations will explore associations between app engagement and mental health outcomes. Exploratory analyses will investigate potential moderators, including demographics (e.g., gender and country of origin) and emotion regulation, for the primary and secondary outcomes, as well as for the engagement and acceptability measures. Sensitivity analyses will be conducted to assess whether outcome patterns differ as a function of intervention engagement or baseline stratification levels of suicidal ideation.

Interview data will be analyzed using a rapid qualitative coding analysis, drawing on principles of Framework Analysis [62,63]. This method is well-suited to small samples and time-sensitive evaluations, allowing for systematic and transparent interpretation of participant feedback across key domains [63]. A deductive coding framework will be developed based on the study aims (e.g., app acceptability, usability, engagement, and perceived impact), while remaining open to inductive insights raised by participants. Data will be summarised within NVivo to facilitate cross-case comparisons and identification of common themes and divergent experiences. This approach balances rigour and practicality and is commonly used in applied health and digital mental health research. Analysis will begin once all interviews are completed.

#### 2.10.3 Missing data

All quantitative analyses will follow intention-to-treat principles. Missing data in outcome measures will be handled using maximum likelihood estimation within the generalised linear mixed models (GLMMs), which provide unbiased estimates under the assumption that data are missing at random (MAR) [64]. This approach is consistent with the planned analytic strategy and enables the use of all available data without imputation. Model diagnostics will be used to examine the plausibility of the MAR assumption. If substantial missingness or deviations from MAR are identified, sensitivity analyses (e.g., multiple imputation or pattern-mixture models) may be conducted to evaluate the robustness of findings.

### 2.11 Safety management

A structured safety protocol is in place to monitor and respond to participant distress and suicide risk. Mental health support information, including crisis contacts and interpreter options, is provided multiple times, including in the app, on the control factsheets, survey completion screens, and PICF. At T1, T2, and T3, participants complete the Kessler Psychological Distress Scale (K10) and are asked if they are having thoughts of suicide (YSIS-3). At T2 and T3, they are additionally asked whether the app, factsheets, or surveys made them feel suicidal or want to self-harm. If a participant scores ≥30 on the K10, endorses suicidal ideation, or reports distress caused by study content, they are offered a wellbeing check-in and provided a list of relevant support resources they can contact. If they accept the check-in, a trained team member experienced in suicide risk assessments will review relevant data (e.g. suicidal ideation score from YSIS-3) and conduct a phone-based risk assessment within two business days. Suicide risk will be based on the phone assessment across four levels (none, low, moderate, high/life-threatening), and appropriate responses will be initiated, including emergency services if necessary (See Table 4). A study psychologist will be available to support staff conducting the risk assessments as required. Participants at life-threatening risk will be withdrawn from the study. Participants at other risk levels are reminded of support options and asked whether they wish to continue in the study.

**Table 4:** Suicide risk levels and associated responses.

Risk Level	Definition	Action
None	No current suicidal thoughts or behaviour; participant reports no distress linked to study participation.	No action required beyond standard follow-up and support information.
Low	Passive thoughts of death or fleeting suicidal ideation without intent, plan, or significant distress.	Participant encouraged to access supports (e.g., helplines, psychologist) as required. Check whether they wish to continue in study.
Moderate	Suicidal ideation present with some associated distress, but no immediate plan or intent to act; participant can ensure their safety.	Participant encouraged to access supports (e.g., helplines, psychologist) as required. Check whether they wish to continue in study.
High/Life-Threatening	Active suicidal ideation with a plan, intent, or expressed inability to stay safe.	Initiate emergency response, withdraw participant, consult study psychologist.

## 3 Conclusions

International students are at elevated risk for mental health problems and suicide, yet they face persistent barriers to accessing culturally appropriate and timely support [5,11]. Traditional service models often fall short due to stigma, cost, and cultural and structural access issues [11,12]. Digital interventions offer an opportunity to address these gaps through timely, low-cost, scalable, and adapted solutions designed in partnership with students [[13], [14], [15]].

This randomized controlled trial will assess the effectiveness, acceptability, engagement, and safety of the Bud app in improving suicide-related risk and protective factors, mental health outcomes, and help-seeking behaviours among international students. The internal pilot will ensure the trial is feasible while maximising data collection for the study outcomes. If found effective, Bud has the potential to be disseminated across tertiary institutions, offering a flexible, low-threshold intervention that empowers international students to better manage their mental health and engage with additional support when needed. In doing so, it may contribute to reducing health inequities and strengthening early intervention strategies within a highly diverse and underserved population.

## CRediT authorship contribution statement

**Samuel McKay:** Writing – review & editing, Writing – original draft, Supervision, Resources, Project administration, Methodology, Funding acquisition, Formal analysis, Conceptualization. **Christina Ng:** Writing – review & editing, Project administration, Methodology. **Jennifer Nicholas:** Writing – review & editing, Methodology, Funding acquisition, Conceptualization. **Vivienne Browne:** Writing – review & editing, Methodology, Funding acquisition, Conceptualization. **Gina Chinnery:** Writing – review & editing, Methodology, Funding acquisition, Conceptualization. **Isabella Choi:** Writing – review & editing, Methodology, Funding acquisition, Conceptualization. **Bailey Nation-Ingle:** Writing – review & editing, Methodology, Conceptualization. **Kristal Alison:** Writing – review & editing, Conceptualization. **Ella Perlow:** Writing – review & editing, Conceptualization. **Michelle Lamblin:** Writing – review & editing, Funding acquisition, Conceptualization. **Elise Carrotte:** Writing – review & editing, Resources, Methodology. **Ellie Brown:** Writing – review & editing, Supervision. **Gregory Armstrong:** Writing – review & editing, Funding acquisition, Conceptualization. **Jocelyn I. Meza:** Writing – review & editing, Funding acquisition, Conceptualization. **Madhavan Mani:** Writing – review & editing, Software. **Jo Robinson:** Writing – review & editing, Supervision, Resources, Funding acquisition, Conceptualization.

## Funding

This work was funded by a Study Melbourne Inclusion Program Grant from the Victorian Government Department of Jobs, Skills, Industry and Regions, An Early Career Researcher Grant from the University of Melbourne, and an Innovation Grant from Suicide Prevention Australia Limited. Jo Robinson is funded by a National Health and Medical Research Council Investigator Grant (ID2008460) and a Dame Kate Campbell Fellowship from the University of Melbourne.

## Declaration of competing interest

The authors declare that they have no known competing financial interests or personal relationships that could have appeared to influence the work reported in this paper.

## Data Availability

The data from this study will not be released publicly until the database is closed at the end of the study. The study investigators will have exclusive access to the data until the publication of the results in a journal. Following the publication of the study findings, the dataset used and/or analyzed during the study will be available from the corresponding author upon reasonable request.

## References

[1] World Health Organization (2021). https://apps.who.int/iris/rest/bitstreams/1350975/retrieve.

[2] Mortier P., Cuijpers P., Kiekens G., Auerbach R.P., Demyttenaere K., Green J.G. (2018 Mar 1). The prevalence of suicidal thoughts and behaviours among college students: a meta-analysis. Psychol. Med..

[3] OECD (2023). https://www.oecd-ilibrary.org/education/education-at-a-glance-2023_e13bef63-en.

[4] Maharaj R., Ndwiga D., Chutiyami M. (2024). Mental health and wellbeing of international students in Australia: a systematic review. J. Ment. Health.

[5] Veresova M., Lamblin M., Robinson J., McKay S. (2024). A systematic review and narrative synthesis of prevalence rates, risk and protective factors for suicidal behaviour in international students. Front. Psychiatr..

[6] McKay S., Veresova M., Bailey E., Lamblin M., Robinson J. (2023 Jan 13). Suicide prevention for international students: a scoping review. Int. J. Environ. Res. Publ. Health.

[7] McGregor S. (2023). Findings into death of AHT. Coroners Court Vic.

[8] Jamieson A. (2021). Findings into death of Nguyen Pham Dinh le. Coroners Court Vic.

[9] Clough B.A., Nazareth S.M., Day J.J., Casey L.M. (2019). A comparison of mental health literacy, attitudes, and help-seeking intentions among domestic and international tertiary students. Br. J. Guid. Counsell..

[10] Zhou X., Zhou A.Q., Sun X. (2022 Jul 3). Prevalence of common mental concerns and service utilization among international students studying in the U.S. Counsel. Psychol. Q..

[11] Sanci L., Williams I., Russell M., Chondros P., Duncan A.M., Tarzia L. (2022 Dec 27). Towards a health promoting university: descriptive findings on health, wellbeing and academic performance amongst university students in Australia. BMC Public Health.

[12] Bartholomew T.T., Robbins K.A., Valdivia-Jauregui L., Lockard A.J., Pérez-Rojas A.E., Keum B.T. (2022 Apr). Center effects, therapist effects, and international student clients' drop out from psychotherapy. J. Counsel. Psychol..

[13] McKay S., Meza J.I. (2024). Culturally contextualized suicide prevention for international students: new opportunities for research and practice. Front. Psychol..

[14] Choi I., Mestroni G., Hunt C., Glozier N. (2023 Feb 17). Personalized help-seeking web application for Chinese-speaking international university students: development and usability study. JMIR Form. Res..

[15] Garrido S., Millington C., Cheers D., Boydell K., Schubert E., Meade T. (2019 Nov 13). What works and what doesn't work? A systematic review of digital mental health interventions for depression and anxiety in young people. Front Psychiatry [Internet].

[16] Torok M., Han J., Baker S., Werner-Seidler A., Wong I., Larsen M.E. (2020 Jan 1). Suicide prevention using self-guided digital interventions: a systematic review and meta-analysis of randomised controlled trials. Lancet Digit. Health.

[17] Hill R.M., Picou P., Hussain Z., Vieyra B.A., Perkins K.M. (2023 Jul 24). https://econtent.hogrefe.com/doi/10.1027/0227-5910/a000917.

[18] Smith-Millman M., Bernstein Larraine, Link Natasha, Hoover Sharon, Lever N. (2022 Jun 22). Effectiveness of an online suicide prevention program for college faculty and students. J. Am. Coll. Health.

[19] King C.A., Eisenberg D., Pistorello J., Coryell W., Albucher R.C., Favorite T. (2022). Electronic bridge to mental health for college students: a randomized controlled intervention trial. J. Consult. Clin. Psychol..

[20] Torok M., Han J., McGillivray L., Wong Q., Werner-Seidler A., O'Dea B. (2022 May 31). The effect of a therapeutic smartphone application on suicidal ideation in young adults: findings from a randomized controlled trial in Australia. PLoS Med..

[21] Malloy J., Partridge S.R., Kemper J.A., Braakhuis A., Roy R. (2023 Jan 1). Co-design of digital health interventions with young people: a scoping review. Digit Health.

[22] O'Brien J., Fossey E., Palmer V.J. (2021 Jan 1). A scoping review of the use of co-design methods with culturally and linguistically diverse communities to improve or adapt mental health services. Health Soc. Care Community.

[23] Dalgleish T., Black M., Johnston D., Bevan A. (2020). Transdiagnostic approaches to mental health problems: current status and future directions. J. Consult. Clin. Psychol..

[24] Batterham P.J., Calear A.L., Farrer L., Gulliver A., Kurz E. (2021). Efficacy of a transdiagnostic self-help internet intervention for reducing depression, anxiety, and suicidal ideation in adults: randomized controlled trial. J. Med. Internet Res..

[25] Coppersmith D.D.L., Dempsey W., Kleiman E.M., Bentley K.H., Murphy S.A., Nock M.K. (2022). Just-in-Time adaptive interventions for suicide prevention: promise, challenges, and future directions. Psychiatry.

[26] Kothgassner O.D., Robinson K., Goreis A., Ougrin D., Plener P.L. (2020 May 11). Does treatment method matter? A meta-analysis of the past 20 years of research on therapeutic interventions for self-harm and suicidal ideation in adolescents. Borderline Personal Disord Emot. Dysregulation.

[27] Van Orden K.A., Witte T.K., Cukrowicz K.C., Braithwaite S.R., Selby E.A., Joiner Jr.TE. (2010). The interpersonal theory of suicide. Psychol. Rev..

[28] Umphrey L.R., Sherblom J.C., Swiatkowski P. (2021 Mar). Relationship of Self-Compassion, hope, and emotional control to perceived burdensomeness, thwarted belongingness, and suicidal ideation. Crisis.

[29] Braun M., Till B., Pirkis J., Niederkrotenthaler T. (2023 May 1). Effects of suicide prevention videos developed by and targeting adolescents: a randomized controlled trial. Eur. Child Adolesc. Psychiatr..

[30] Cai C., Qu D., Liu D., Liu B., Zhang X., Chen P. (2023 Nov 1). Effectiveness of a localised and systematically developed gatekeeper training program in preventing suicide among Chinese adolescents. Asian J. Psychiatr..

[31] Roslan A.F., Pheh K.S., Mahadevan R., Bujang S.M., Subramaniam P., Yahya H.F. (2023 Jan 19). Effectiveness of online advanced C.A.R.E suicide prevention gatekeeper training program among healthcare lecturers and workers in national university of Malaysia: a pilot study. Front Psychiatry [Internet].

[32] Skromanis S., Cooling N., Rodgers B., Purton T., Fan F., Bridgman H. (2018 Jun). Health and well-being of international university students, and comparison with domestic students, in Tasmania, Australia. Int. J. Environ. Res. Publ. Health.

[33] Xiong W., Quinney B., King D.L., Ali K., Radunz M., Zhao Y. (2025 Jul 1). Cross-sectional study investigating the mental health and wellbeing of university students in Australia. Int. J. Intercult. Relat..

[34] Chamberlain P., Goldney R., Delfabbro P., Gill T., Dal Grande L. (2009 Jan). Suicidal ideation: the clinical utility of the K10. Crisis.

[35] Dingwall K.M., Povey J., Sweet M., Friel J., Shand F., Titov N. (2023 Jun 7). Feasibility and acceptability of the Aboriginal and islander mental health initiative for Youth app: nonrandomized pilot with first nations young people. JMIR Hum Factors.

[36] Dear B.F., Fogliati V.J., Fogliati R., Johnson B., Boyle O., Karin E. (2018 Jul 1). Treating anxiety and depression in young adults: a randomised controlled trial comparing clinician-guided versus self-guided internet-delivered cognitive behavioural therapy. Aust. N. Z. J. Psychiatr..

[37] Kirshenbaum J.S., Pagliaccio D., Bitran A., Xu E., Auerbach R.P. (2024 Jun 27). Why do adolescents attempt suicide? Insights from leading ideation-to-action suicide theories: a systematic review. Transl. Psychiatry.

[38] Servaty‐Seib H.L., Lockman J., Shemwell D., Reid Marks L., Servaty-Seib H.L., Lockman J., Arria Bryan, Cheng Cohen, Conner Cukrowicz, Davis DeBard, Drum Field, Glass Hanassab, Hayes Hess, Howe Huebner, Joiner Joiner, Joiner Konick, Lamis Maundeni, Mazza Osterman, Schwartz Van Orden, Orden Van, Orden Van, Orden Van, Orden Van, Wang B. (2016 Apr). International and domestic students, perceived burdensomeness, belongingness, and suicidal ideation. Suicide Life-Threatening Behav..

[39] Han R., Walter Z.C., Maccallum F., Dingle G.A. (2024 Oct 16). First-year domestic and international university students' knowledge and use of mental health services and self-care practices in Australia. Aust. Psychol..

[40] Colmenero-Navarrete L., García-Sancho Esperanza, Salguero J.M. (2022 Oct 2). Relationship between emotion regulation and suicide ideation and attempt in adults and adolescents: a systematic review. Arch. Suicide Res..

[41] Green P., MacLeod C.J. (2016). SIMR: an R package for power analysis of generalized linear mixed models by simulation. Methods Ecol. Evol..

[42] Bennett S., Myles-Hooton P., Schleider J., Shafran R. (2022).

[43] McKay M., Wood J.C., Brantley J. (2019). Emotion Regulation, and Distress Tolerance.

[44] Webb M., Carrotte E.R., Flego A., Vincent B., Lee-Bates B., Heath J. (2023 May). Safety, acceptability, and initial effectiveness of a novel digital suicide prevention campaign challenging perceived burdensomeness. Crisis.

[45] Raes F., Pommier E., Neff K.D., Van Gucht D. (2011). Construction and factorial validation of a short form of the self-compassion scale. Clin. Psychol. Psychother..

[46] Harris P.A., Taylor R., Thielke R., Payne J., Gonzalez N., Conde J.G. (2009 Apr 1). Research electronic data capture (REDCap)—A metadata-driven methodology and workflow process for providing translational research informatics support. J. Biomed. Inf..

[47] Harris P.A., Taylor R., Minor B.L., Elliott V., Fernandez M., O'Neal L. (2019 Jul). The REDCap consortium: building an international community of software platform partners. J. Biomed. Inf..

[48] O'Donnell M.B., Shirley L.A., Park S.S., Nolen J.P., Gibbons A.M., Rosén L.A. (2018). The college adjustment questionnaire: a measure of students' educational, relational, and psychological adjustment to the college environment. J. Coll. Student Dev..

[49] Felitti V.J., Anda R.F., Nordenberg D., Williamson D.F., Spitz A.M., Edwards V. (1998 May 1). Relationship of childhood abuse and household dysfunction to many of the leading causes of death in adults: the Adverse Childhood Experiences (ACE) study. Am. J. Prev. Med..

[50] Kessler R.C., Andrews G., Colpe L.J., Hiripi E., Mroczek D.K., Normand S.L.T. (2002 Aug). Short screening scales to monitor population prevalences and trends in non-specific psychological distress. Psychol. Med..

[51] Deane F.P., Wilson C.J. (2005). Considerations for specifying problem-types, help-sources and scoring the General Help-seeking Questionnaire (GHSQ). Can. J. Counsell..

[52] Bryan C.J., Clemans T.A., Hernandez A.M. (2012 Feb 1). Perceived burdensomeness, fearlessness of death, and suicidality among deployed military personnel. Pers. Indiv. Differ..

[53] Kaufman E.A., Xia M., Fosco G., Yaptangco M., Skidmore C.R., Crowell S.E. (2016 Sep 1). The difficulties in emotion regulation scale short form (DERS-SF): validation and replication in adolescent and adult samples. J. Psychopathol. Behav. Assess..

[54] Bjørnsen H.N., Bjørnebekk G., Brandmo C. (2024 May 1). Schools as a source of mental health literacy: adjusting and validating a mental health literacy scale. Health Promot. Pract..

[55] Stoyanov S.R., Hides L., Kavanagh D.J., Wilson H. (2016 Jun 10). Development and validation of the user version of the Mobile Application Rating Scale (uMARS). JMIR mHealth uHealth.

[56] Wojujutari A.K., Idemudia E.S. (2024). Consistency as the currency in psychological measures: a reliability generalization meta-analysis of kessler psychological distress Scale (K-10 and K-6). Depress. Anxiety.

[57] Hetrick S.E., Gao C.X., Filia K.M., Menssink J.M., Rickwood D.J., Herrman H. (2021 Dec 1). Validation of a brief tool to assess and monitor suicidal ideation: the Youth Suicide Ideation Screen (YSIS-3). J. Affect. Disord..

[58] Byrne S.J., Bailey E., Lamblin M., Pirkis J., Mihalopoulos C., Spittall M.J. (2021). Study protocol for the Multimodal Approach to Preventing Suicide in Schools (MAPSS) project : a regionally-based trial of an integrated response to suicide risk among secondary school students. BMC Trials.

[59] Avery K.N.L., Williamson P.R., Gamble C., Francischetto E.O., Metcalfe C., Davidson P. (2017 Feb 1). Informing efficient randomised controlled trials: exploration of challenges in developing progression criteria for internal pilot studies. BMJ Open.

[60] Kasckow J., Youk A., Anderson S.J., Dew M.A., Butters M.A., Marron M.M. (2016 Feb 1). Trajectories of suicidal ideation in depressed older adults undergoing antidepressant treatment. J. Psychiatr. Res..

[61] Adrian M., Miller A.B., McCauley E., Vander Stoep A. (2016). Suicidal ideation in early to middle adolescence: sex-specific trajectories and predictors. JCPP (J. Child Psychol. Psychiatry).

[62] Parkinson S., Eatough Virginia, Holmes Joshua, Stapley Emily, Midgley N. (2016 Apr 2). Framework analysis: a worked example of a study exploring young people's experiences of depression. Qual. Res. Psychol..

[63] Gale N.K., Heath G., Cameron E., Rashid S., Redwood S. (2013 Sep 18). Using the framework method for the analysis of qualitative data in multi-disciplinary health research. BMC Med. Res. Methodol..

[64] Enders C.K. (2010).

